# Effects of the *SLCO1B1* A388G single nucleotide polymorphism on the development, clinical parameters, treatment, and survival of multiple myeloma cases in a Polish population

**DOI:** 10.1007/s11033-022-08162-x

**Published:** 2022-12-07

**Authors:** Katarzyna Michalska, Ewa Balcerczak, Agnieszka Jeleń, Lias Saed, Jacek Pietrzak, Marta Żebrowska-Nawrocka

**Affiliations:** 1 Laboratory of Molecular Diagnostics and Pharmacogenomics, Department of Pharmaceutical Biochemistry and Molecular Diagnostics, Medical University of Lodz, Muszynskiego 1, 90-151 Lodz, Poland; 2 Laboratory of Molecular Diagnostics and Pharmacogenomics, Department of Pharmaceutical Biochemistry and Molecular Diagnostics, Interfaculty Cathedral of Laboratory and Molecular Diagnostics, Medical University of Lodz, Muszynskiego 1, 90-151 Lodz, Poland

**Keywords:** Multiple myeloma, *SLCO1B1*, OATP, SNP, Survival, Drug

## Abstract

**Background:**

Multiple myeloma is one of the most common hematological malignancies worldwide. Genetic alterations may lead to the progression from monoclonal gammopathy to multiple myeloma. Additionally, the genetic background of the disease might influence therapy outcomes, including survival time. SLCO1B1, belonging to the OATPs family, is a membrane protein that mediates the uptake of a wide range of endogenous and exogenous (including drugs) compounds.

**Methods and results:**

In this study, the A388G single nucleotide polymorphism in the *SLCO1B1* gene in Polish multiple myeloma patients was determined. This polymorphism affects the amino acid change of the protein, so it may be responsible for treatment effectiveness or risk of disease development. A388G was evaluated by the PCR–RFLP method. The presented study showed a statistically significant association between the GG genotype with longer survival of patients with multiple myeloma with Melphalan-Prednisone therapy compared to other treatment regimens (*p* = 0.0271). There was no statistically significant association in the frequency of genotypes (*p* = 0.8211) and alleles: allele A (*p* = 0.5442); allele G (*p* = 0.8020) between multiple myeloma patients and a control group.

**Conclusions:**

The A388G polymorphism does not seem to affect the increased risk of the development of multiple myeloma. However, the occurrence of the GG genotype may prolong of patients overall survival in the case of Melphalan-Prednisone therapy.

## Background

Multiple myeloma (MM) is a malignant plasma cell disorder characterized by the clonal proliferation of plasma cells in bone marrow [1–3]. MM is one of the most common hematological malignancies in adults worldwide and accounts for 1.8% of all cancer cases and approximately 10% of hematologic malignancies [3, 4]. In Europe, more than 48,000 new cases and approximately 31,000 deaths are reported each year from multiple myeloma [5, 6].

Multistep genetic alterations lead to the progression from MGUS—monoclonal gammopathy of undetermined significance to multiple myeloma in some persons. The monoclonal protein produced by plasma cells is an abnormal immunoglobulin either of which causes excessive viscosity and end-organ damage. The most common type of heavy chain produced in myeloma is IgG, followed by IgA, then IgD and light chain only. IgM, and IgE are very rare. The MM presents with hypercalcaemia, renal failure, anemia and bone lesions, collectively known as CRAB criteria. The most common abnormality in basic laboratory tests results is anemia, i.e. a reduced number of red blood cells or reduced amount of hemoglobin they contain. Similarly, elevated creatinine levels, indicative of impaired kidney function as a result of damage by monoclonal proteins, have been observed frequently [7].

Over the past two decades, strategies for MM therapy have rapidly evolved and led to improved clinical outcomes including prolonged survival, but predicting the treatment response of individual patients at the time of diagnosis remains difficult and needs an understanding of genetic background [8]. This could contribute to a more complete molecular description of the disease and improved therapy. In that context study of membrane transporters may have great clinical importance, because to function properly, the human body has to constantly transport various substances, both xenobiotics, and endogenous compounds, through biological barriers.

There are many groups of membrane transporters divided into families due to the homology of structure and the specifics of transported substrates. In humans, the solute carrier (SLC) family of membrane transport proteins consists of approximately 300 individual proteins and is organized into 43 families [9]. The organic anion transporting polypeptide (OATP) superfamily includes important transporters which substrates are many endogenous and xenobiotic substances, including: bile acids, organic pigments, nucleotides, prostaglandins, hormone precursors, hormones, environmental toxins, and anticancer drugs. Therefore, OATPs are capable to transport a wide variety of compounds affecting growth and survival of both normal and cancer cells [10–13].

The pharmaceutical substances transported by OATP include: antibiotics, antidiabetic drugs, anti-inflammatory drugs, antifungals, antivirals, antihistamines, antihypertensives, fibrates, statins, cardiac glycosides, immunosuppressants, anticancer drugs, including those used in the treatment of multiple myeloma *e.g*. methotrexate, doxorubicin, and docetaxel [9, 10, 14–16]. OATPs as the main of influx drug transporters significantly contribute to the absorption, distribution, and elimination (ADME) of pharmaceutical agents and the involvement of drug-drug interaction (DDI). The expression of OATPs transporters in neoplasms may also influence the intracellular concentration of drugs, thus influencing their effectiveness [9, 14, 17, 18].

OATPs are expressed in various tissues and organs, such as the liver, intestine, blood–brain barrier, kidney, placenta, and other organs [9, 19, 20]. It is now well recognized, that certain OATPs are differentially regulated in normal and cancer tissues [20]. There is evidence that the expression of some OATPs may be varied in several types of cancers, suggesting their potential pathogenic roles during the development and progression of cancer [10, 21]. OATPs expression levels have been shown to be altered in many different types of cancer, and some are correlated with cancer stage and outcomes. So far, research on the role of OATP1B1 has focused on their role in pharmacokinetics, treatment efficacy, and clinical outcomes in the treatment of solid tumors. So far, less research has been done on the role of these transporters in hematological malignancies [22–24].

Several of naturally occurring single nucleotide polymorphisms (SNPs) in the genes encoding OATPs have been reported and extensively investigated for their impact on the expression and function of OATP transporters. Many studies have shown that SNPs from OATP are associated with an effect on the presence and function of proteins, and that some SNPs are associated with an altered distribution of chemotherapy drugs and, consequently, with increased side effects [12]. So far, it has been reported that polymorphic variants of the genes encoding OATP1A2, OATP1B1, and OATP1B3 are clinically significant [10].

The *SLCO1B1* gene located on chromosome 12 (gene *locus* 12p12) consists of 15 exons and 14 introns, and encodes a protein with 691 amino acids [25, 26]. The *SLCO1B1* gene spans 15 exons and 190 common variants with minor allele frequency, greater than 5% [26, 27]. Although many SNPs have been identified in *SLCO1B1*, only several are known to have functional effects and clinical significance, e.g*. SLCO1B1* rs2306283 (A388G*,* N130D) or rs4149056 (T521C, V174A) [16, 26, 28]. Differently regulated OATPs may play a pathogenic role in cancer development or progression, and potentially serve as therapeutic targets for cancer [29]. SNP A388G (rs2306283) has been reported to affect the transport function of the protein, which is due to changes in the structure of transmembrane domains, but the functional consequences of this variant remain controversial [28, 30, 31].

Our work aimed to determine *SLCO1B1* A388G gene polymorphism in multiple myeloma patients. The research may allow us to better understand the molecular mechanisms underlying the altered function, expression of OATPs in cancer development, transport of anticancer drugs, and therapy efficiency, to try to use the assessment of transporters’ status as potential molecular markers of the diagnostic, prognostic, and predictive nature or in cancer treatment involving individual response to drugs.

## Material and method

The investigated group: 157 blood samples were collected from patients with multiple myeloma diagnosed at the Department of Hematology, Medical University of Lodz, Poland between 1992 and 2002 according to the International Myeloma Working Group Classification criteria. Due to the lack of complete clinicopathological data for all patients, some statistical analyzes were performed on less numerous groups.

The control group: 141 blood samples were obtained from healthy individuals from the local blood bank, who geographically and ethnically matched the group of patients with MM.

The investigation was carried out in accordance with the principles of the Declaration of Helsinki and was approved by the Ethical Committee of the Medical University of Lodz No: RNN/93/20/KE, RNN/88/16/KE; RNN/285/13/KE. Informed consent was obtained from all individual participants included in the study.

### DNA isolation

The DNA was isolated from peripheral blood using the microcolumn method under the "Blood Mini" protocol (*A&A Biotechnology, Poland*). The DNA samples, until further analysis, were stored at − 20 °C. The DNA quantity and quality/purity were determined by Nanophotometer *(IMPLEN, Germany)*.

### Genotyping of A388G

To determine the A388G polymorphism in the *SLCO1B1* gene, the PCR‐restriction fragment length polymorphism method (PCR‐RFLP) was used. For analyzing the *SLCO1B1* variants, the forward primer 5′‐CATGCTGGGAAATTGACAGAAAGT‐3′ and the reverse primer 5′‐GAAAACGCGTAGTTTAAACCTGT ‐3′ were used. The PCR reaction was performed in a total reaction volume of 20 μl volume containing: 50 ng of genomic DNA, 10 μl of REDTaq® ReadyMix™ (*Sigma- Aldrich, USA*), 0.7 μl of 10 µmol of forward and reverse primers, and distilled water up to a final volume. A negative control was included in each experiment. The PCR product for the A388G SNP of the *SLCO1B1* gene was 462 bp in size. In the next step, PCR products were digested with the *TaqI* restriction enzyme (*EURX Sp. z o. o., Poland*) at 65 °C for 16 h. The digested PCR products were separated by electrophoresis using a 3% agarose gel stained with ethidium bromide and visualized by a UV transilluminator. Electrophoretic analysis of genotypes was performed. The bands patterns presentation was: AA (*wild-type*) 194 + 268, AG (*heterozygote*) 23 + 171 + 194 + 268, GG (*mutant*) 23 + 171 + 268.

### Statistical analysis

The statistical analyses were performed using STATISTICA 13 software *(StatSoft Inc. 2018).* To determine differences between genotype and allele frequencies of A388G among multiple myeloma patients and the control group as well as the significance of differences A388G polymorphism and clinical–pathological features of the MM patients the chi‐square test was used. The Kaplan–Meier analysis was performed to estimate overall survival (OS). The OS was defined as the interval from the date of diagnosis to the date of death or the last clinical appointment. The effects of A388G polymorphism on survival were examined using the chi‐square test for genotypes and log-rank test for alleles using the proportional hazards model. In all conducted tests, a *p *value of < 0.05 was considered significant.

### In silico analysis

The effect of A388G polymorphism on the protein was estimated by in silico programs:

- SIFT (Sorting Intolerant From Tolerant; https://sift.bii.a-star.edu.sg/www/SIFT_dbSNP.html).
- PolyPhen-2 (http://genetics.bwh.harvard.edu/pph2/).
- PROVEAN (Protein Variation Effect Analyzer; http://provean.jcvi.org/index.php).
- Mutation Taster (https://www.mutationtaster.org/MutationTaster69/index.html).

## Results

In our results, similarly to Caucasians, the *SLCO1B1* in A388G SNP G allele was observed with a lower frequency in the Polish population [11, 26, 28, 32]. The frequencies of alleles and genotypes of the studied A388G SNP between the investigated and control groups were compared. There were no statistically significant differences in the frequencies of the genotype (*p* = 0.8211) and alleles: A (*p* = 0.5442) and G (*p* = 0.8020) between groups. However, the GG genotype occurred slightly more often in the group of MM patients than in the control group (28.7%; 25.5%, respectively). The GG genotype was associated with a 1.15-fold higher incidence of this disease compared to the AG and AA genotypes. The details are presented in Table 1.

**Table 1:** Frequency of genotype and allele distributions of the A388G SNP between the investigated and control group

SLCO1B1 SNP A388G		Multiple myeloma N = 157 [%]	Healthy individuals N = 141 [%]	*p*	OR [95%]
Genotype	AA	79 [50.3]	73 [51.8]	0.8211	1
	AG	33 [21.0]	32 [22.7]		0.95 [0.53–1.70]
	GG	45 [28.7]	36 [25.5]		1.15 [0.67–1.98]
Allele^a^	A present	191 [60.8]	178 [63.1]	0.5442	–
	A absent	123 [39.2]	104 [36.9]		
	G present	123 [39.2]	104 [36.9]	0.8020	–
	G absent	191 [60.8]	178 [63.1]		

^a^Total N for present/absent alleles calculated as the sum of present/absent alleles in each genotypes

The reduced number of cases used in further statistical analyzes resulted from the limited availability of clinical data. Among the group of 97 MM patients, 47 were men (48.5%) and 50 were women (51.5%). No statistically significant differences were found in the distribution of genotypes (*p* = 0.9147) and the frequency of alleles A allele or the G allele (*p* = 0.6738 and *p* = 0.7813, respectively) depending on the patient gender.

The next compared parameter was age. The median age of MM patients at diagnosis was 63 years (N = 79; age range 40 to 87 years). The patients were divided into two subgroups: the first subgroup of patients aged ≤ 63 years and the second subgroup aged over 63 years. The genotype AA tended to occur more frequently in the subgroup of patients aged ≤ 63 years (*p* = 0.0742). The allele analysis showed that the occurrence of at least one A allele was statistically significantly more frequent (*p* = 0.0357) in the subgroup of patients under 63 years of age (79%) than in the subgroup over 63 years (58%).

Many laboratory parameters allow clinicians to monitor the disease progress, assessing the effectiveness of treatment and prognosis in multiple myeloma. The most common abnormality in basic laboratory test results is anemia, i.e. a reduced a number of red blood cells or reduced amount of hemoglobin they contain. Similarly, elevated creatinine levels, indicative of impaired kidney function as a result of damage by monoclonal proteins, have been observed frequently. In the next stage of the analysis, the group of patients with MM for whom clinical trials were available were divided into subgroups according to hemoglobin and creatinine levels (N = 61 and N = 59, respectively). In the case of the hemoglobin level (Hb), the first subgroup of patients had it lower than or equal to 9.2 g/dL, the second subgroup had the Hb level over 9.2 g/dL; and as regards the creatinine concentration, the first subgroup had it lower than or equal to 2 mg/dL, the second subgroup had this level over 2 mg/dL. When we compared the distribution of the A388G SNP according to the hemoglobin level in the subgroups of MM patients, no significant differences for investigated genotypes and alleles were observed (*p* = 0.2020). However, the A allele tended to be more frequent in the subgroup of patients with a hemoglobin level lower or equal to 9.2 g/dL (*p* = 0.0771). In the case of the creatinine concentration analysis, no statistical significance was demonstrated (*p* = 0.7133).

Further analysis according to the stage of advancement of MM in line with the Durie-Salmon classification was performed (N = 52). In this part of the analysis also no significant differences were observed in the genotype frequencies (*p* = 0.2075). It is worth noting that at least one A allele was more frequent in the subgroup of patients who were classified as stage III (80%) than in stage I (60%) or stage II (43%) according to the Durie-Salmon classification (*p* = 0.0974). The distributions of genotype and allele frequencies of the analyzed clinical-pathological features are summarized in Table 2.

**Table 2:** The frequency of the studied A388G SNP in patients with multiple myeloma according to the clinical-pathological features

		N	Prevalence of the investigated A388G SNP in multiple myeloma patients									
			AA [%]	AG [%]	GG [%]	*p*	Apresent [%]	Aabsent [%]	*p*	Gpresent [%]	Gabsent [%]	*p*
Gender	Female	50	28 [56]	8 [16]	14 [28]	0.9147	36 [72]	14 [28]	0.6738	22 [44]	28 [56]	0.7813
	Male	47	25 [53]	7 [15]	15 [32]		32 [68]	15 [32]		22 [47]	25 [53]	
Age	≤ 63 years	39	26 [67]	5 [12]	8 [21]	0.0742	31 [79]	8 [21]	*0.0357*	13 [33]	26 [67]	*0.0311*
	> 63 years	40	17 [43]	6 [14]	17 [43]		23 [58]	17 [42]		23 [58]	17 [42]	
Hemoglobin	≤ 9.2 g/dL	31	20 [65]	5 [16]	6 [19]	0.2020	25 [80]	6 [20]	0.0771	11 [34]	20 [65]	0.2517
	> 9.2 g/dL	30	15 [50]	3 [10]	12 [40]		18 [60]	12 [40]		15 [50]	15 [50]	
Stage of advancement according to Durie-Salmon	I	5	3 [60]	0 [0]	2 [40]	0.2075	3 [60]	2 [40]	0.0974	2 [40]	3 [60]	0.6203
	II	7	3 [43]	0 [0]	4 [57]		3 [43]	4 [57]		4 [57]	3 [43]	
	III	40	25 [63]	7 [17]	8 [20]		32 [80]	8 [20]		15 [37]	25 [63]	
Creatinine ≥ 2 mg/dL	No	44	24 [55]	6 [13]	14 [32]	0.7689	30 [68]	14 [32]	0.9136	20 [45]	24 [55]	0.7133
	Yes	15	9 [60]	1 [7]	5 [33]		10 [67]	5 [33]		6 [40]	9 [60]	
Type of chemotherapy	MP	28	12 [43]	6 [21]	10 [36]	0.6707	18 [64]	10 [36]	0.3574	16 [57]	12 [43]	0.4569
	VAD	41	23 [56]	8 [20]	10 [24]		31 [78]	10 [22]		18 [44]	23 [56]	
	Other	8	5 [63]	1 [17]	2 [25]		7 [88]	1 [12]		3 [37]	5 [63]	

*MP *Melphalan-Prednisone, *VAD* Vincristine-Adriamycin-Dexamethasone, *Other* Melphalan/or Bortezomib/or Bortezomib + VAD

The type of myeloma diagnosis does not usually influence treatment, but it can affect the course of the disease in an individual patient. Clinical data on the type of immunoglobulins secreted by myeloma cells were available for 78 trials. The group of patients with MM was divided into three subgroups according to the type of the produced immunoglobulins. The produced immunoglobulin subtype was IgG for 46 patients (59%), IgA for 17 patients (22%), and light chains for 15 patients (19%). Also, in this case, no statistical association was found between the different genotypes and alleles of the A388G SNP of the *SLCO1B1* gene and the type of produced immunoglobulins (*p* = 0.6939). Details are in in Table 3.

**Table 3:** The frequency of genotypes and alleles of SNP A388G in the *SLCO1B1* gene in patients with multiple myeloma in relation to the type of immunoglobulins secreted by myeloma cells

SLCO1B1 SNP A388G		Multiple myeloma patients (N = 78)			*p*
		Immunoglobulin subtype			
		IgG [%]	IgA [%]	Light chains [%]	
Genotype	AA	27 [34.6]	7 [9.0]	8 [10.2]	0.6939
	AG	14 [17.9]	6 [7.7]	5 [6.4]	
	GG	5 [6.4]	4 [5.1]	2 [2.6]	
Allele*	A present	32 [41.1]	11 [14.1]	10 [12.8]	0.9284
	A absent	14 [17.9]	6 [7.7]	5 [6.4]	
	G present	19 [24.3]	10 [12.8]	7 [9.0]	0.4642
	A absent	27 [34.7]	7 [9.0]	8 [10.2]	

*Total N for present/absent alleles calculated as the sum of present/absent alleles in each genotypes

**Fig. 1:**
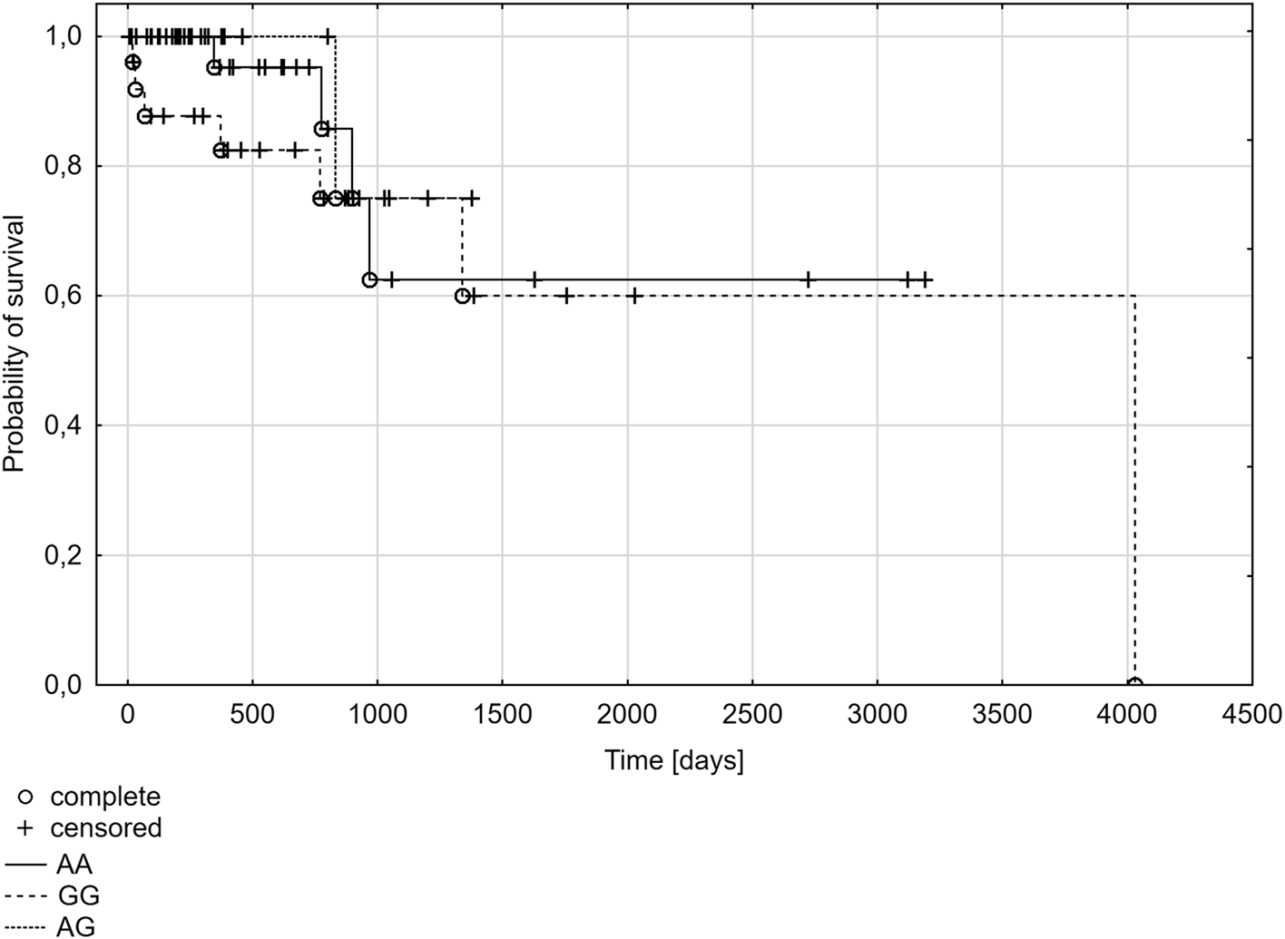
Kaplan–Meier plot for multiple myeloma patients with different genotypes for A388G polymorphism of the *SLCO1B1* gene

**Fig. 2:**
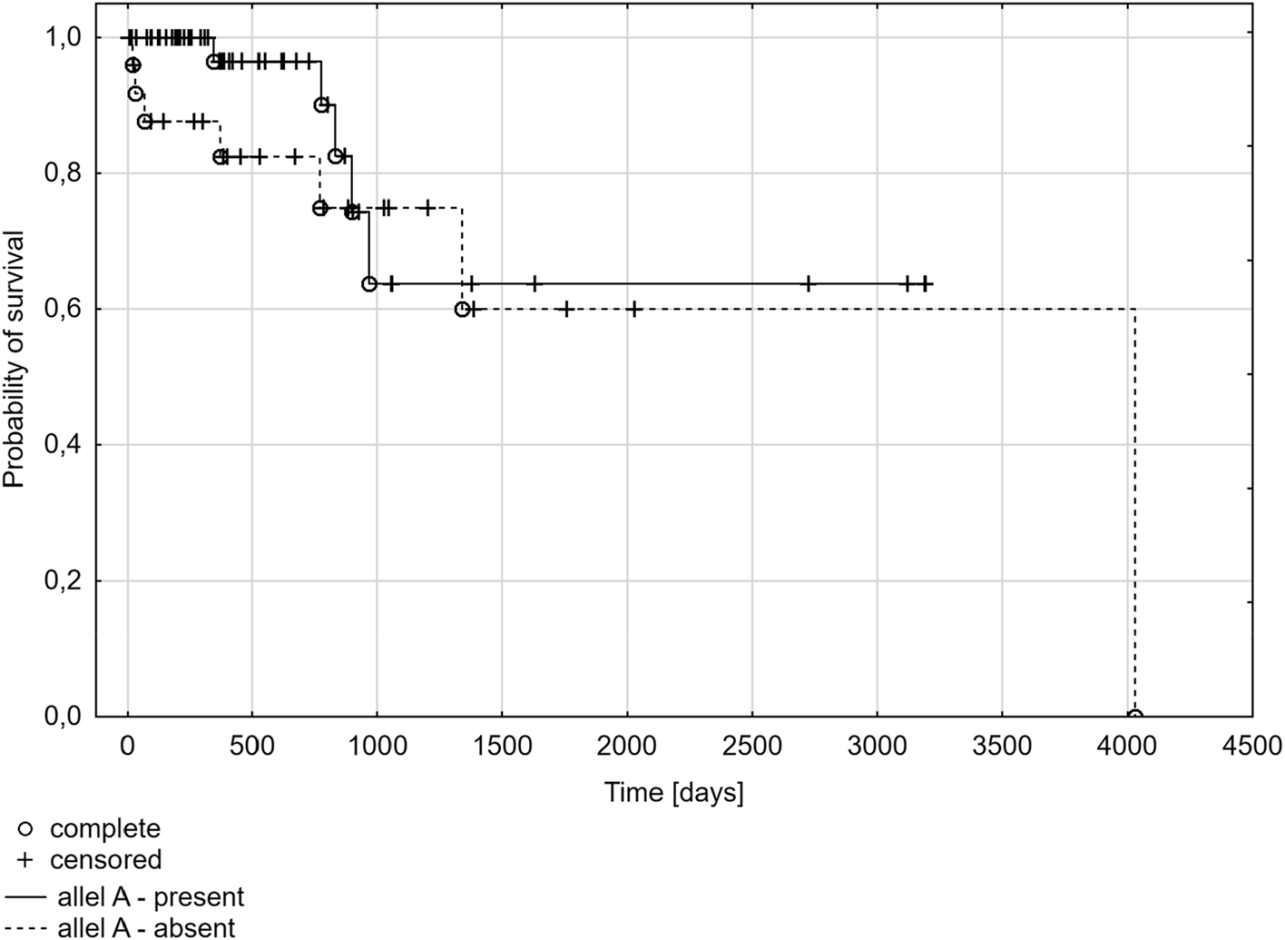
Kaplan–Meier plot for multiple myeloma patients with the present/ absent A allele in the A388G polymorphism of the *SLCO1B1* gene

**Fig. 3:**
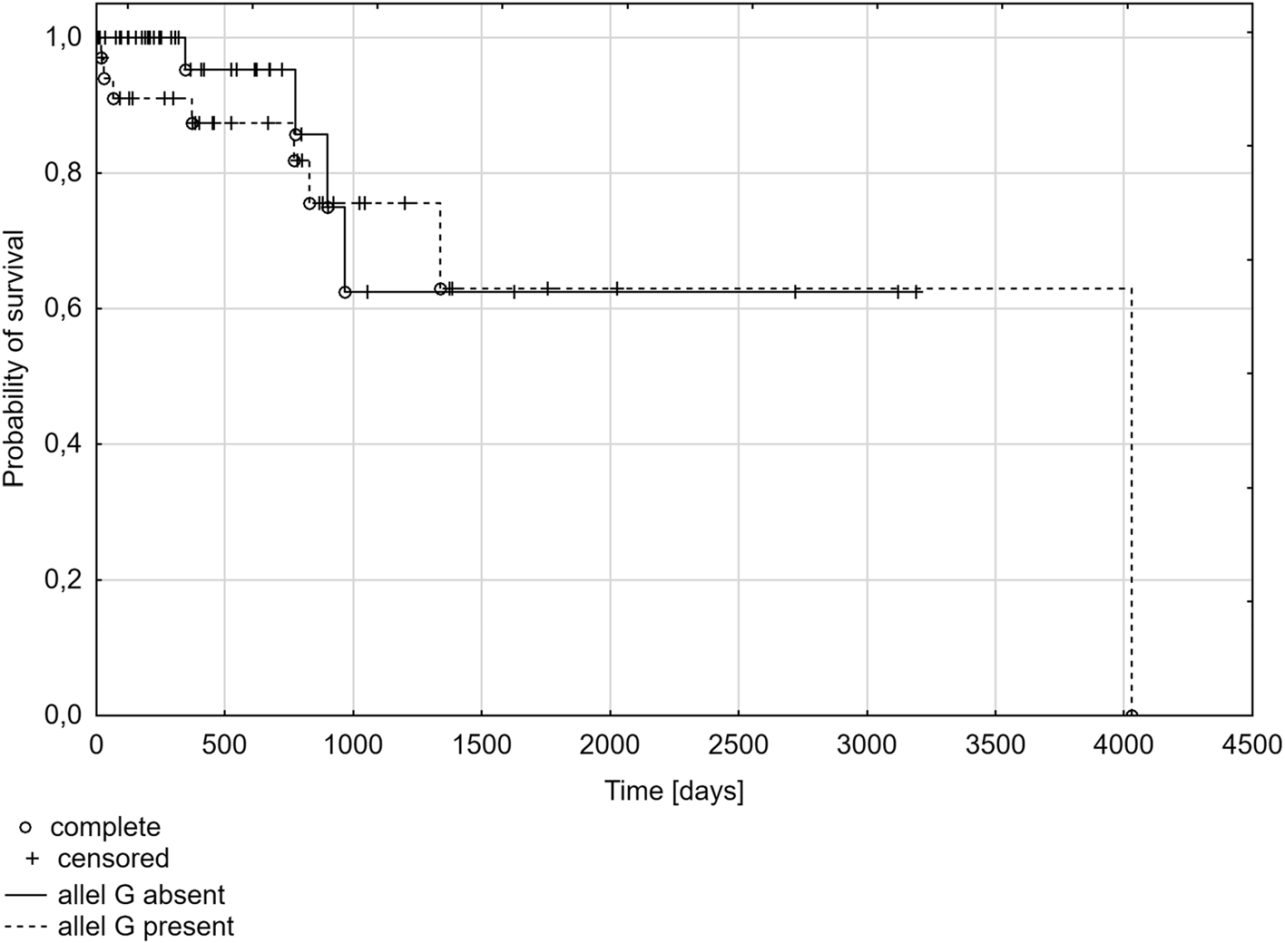
Kaplan–Meier plot for multiple myeloma patients with the present/absent G allele in the A388G polymorphism of the *SLCO1B1* gene

The dependence of the polymorphism at position A388G of the *SLCO1B1* gene with the probability of overall survival time was analyzed as the last part. The Kaplan–Meier plot shows the probability of survival in the group of patients with multiple myeloma from the diagnosis to the last follow-up. There was no statistically significant difference in survival according to genotypes (*p* = 0.1192) or the presence of at least one A or G allele (*p* = 0.3122, *p* = 0.5587*,* respectively). However, the time of survival was shorter in the subgroup of patients with the AA genotype (median: 321 days) compared to the subgroups of patients with the GG genotype (median: 628 days) or the AG genotype (median: 526 days) (Fig. 1). This is confirmed by the results of the analysis for A388G SNP alleles, where the time of survival was shorter in the presence of at least one A allele (A allele present: median 379 days; A allele absent: median 526 days) (Fig. 2), and it was longer in the presence of at least one G allele (G allele present: median 597 days; G allele absent: median 321 days) (Fig. 3).

**Fig. 4:**
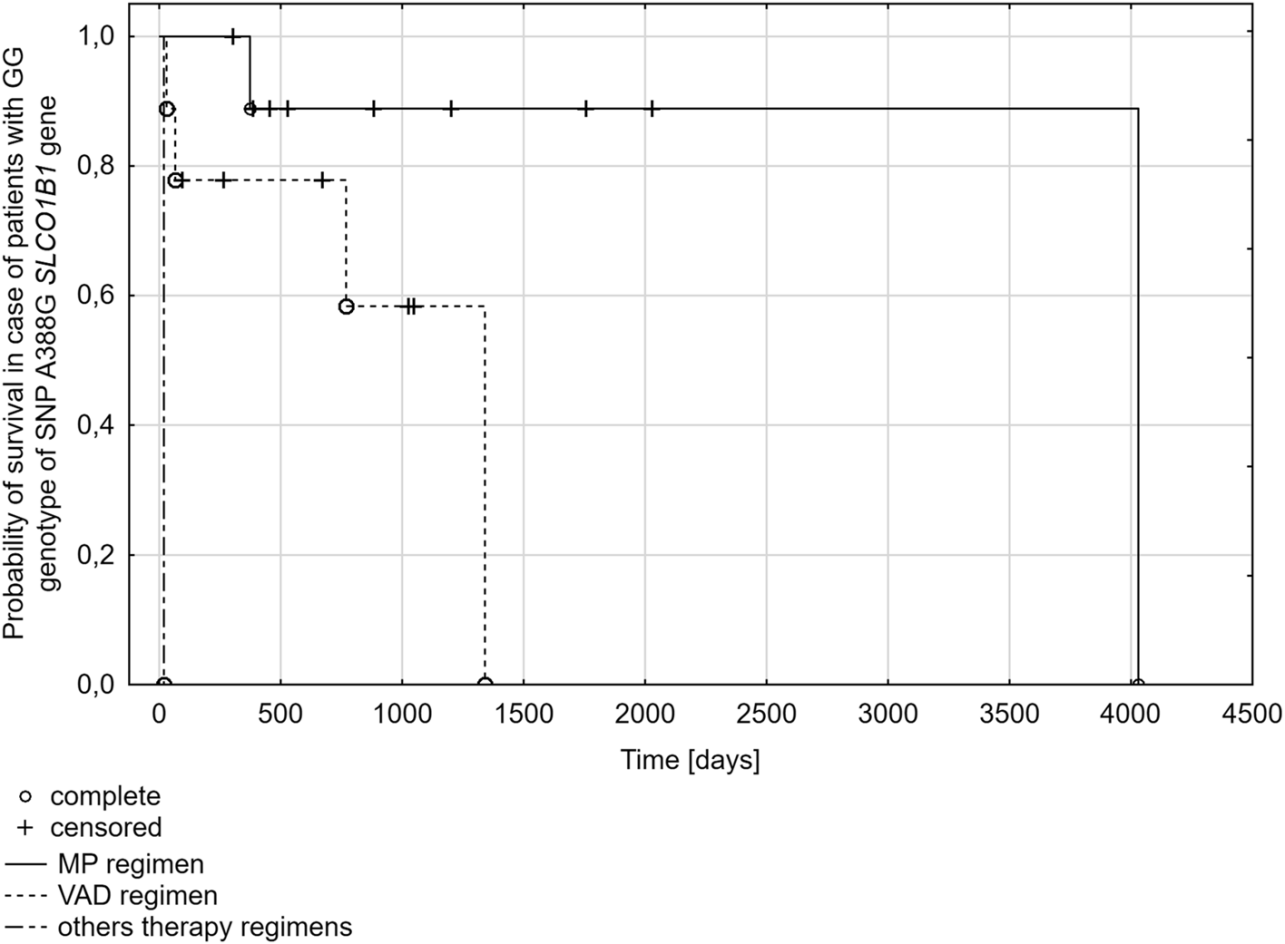
Kaplan–Meier plot for multiple myeloma patients with GG genotype for A388G polymorphism of the *SLCO1B1* gene according to the different treatment regimen

The data on the type of scheme of chemotherapy used during the treatment was available for 77 MM patients. The Melphalan-Prednisone—MP scheme was applied in 28 (36%) cases, Vincristine-Adriamycin-Dexamethasone—VAD scheme was used in 41 (53%) cases. Different treatment regimens were used in the remaining cases 8 (11%). Patients with MM for whom information about the treatment was available were divided according to the A388G genotype and in these subgroups, the influence of the applied treatment regimen on survival was assessed. In the group of patients with the GG genotype, the survival time was statistically significantly longer (*p* = 0.0271) in the case of the MP (704 days) regimen compared to the VAD regimen (669 days) or other regimens (3 days) (Fig. 4). No statistical significance was demonstrated in the analysis concerning the association between the A388G SNP and the treatment scheme according to genotypes (*p* = 0.6707) or the presence of at least one A or G allele (*p* = 0.3574, *p* = 0.4569, respectively). When the effect of the treatment regimen on survival was assessed, it was shown that the longest median survival (471 days) was when the VAD regimen was applied compared to the MP regimen (451 days) and other treatments (95 days). However, it was not statistically significant (*p* = 0.1171).

Due to contradictory reports on the importance of this polymorphism on the amount and function of the protein as the last part, an in silico analysis was performed for assessing the potential impact of investigated SNP on encoded protein function. All analyses showed that A388G polymorphism does not affect on SLCO1B1 protein structure and function. Details are in Table 4.

**Table 4:** In silico evaluation on the effect of nonsynonymous SNP A388G of *SLCO1B1* gene

SNP	TOOL	SCORE	PREDICTION
A388G	SIFT	0.561	Tolerated
	PolyPhen-2	0.000	Benign
	PROVEAN	−1.784	Neutral
	Mutation Taster	23	Probably harmless

## Discussion

OATPs are membrane proteins that mediate the uptake of a wide range of endogenous compounds and many xenobiotics, thus ensuring the regulation of delivery of required substrates and thereby cellular homeostasis [18, 19]. Changes in the amount and / or activity of transport proteins have numerous consequences, e.g. they affect the cell defense potential by regulating the amount of harmful substances in the cell, which may lead to cell damage, mutation, and oncogenesis. So far, it has been shown that the expression of some OATPs can be altered in a variety of disease states, including many different types of cancer. OATPs have been found to be alter expressed in a variety of human solid tumors, including breast, liver, colon, pancreatic, and ovarian cancers, but a smaller number of analyzes concerns hematological cancers. Additionally, the level of protein activity is usually related to the response to the chemotherapy administered [10, 12, 17, 20, 28, 33–35].

In several cancers, altered expression of OATP levels has been correlated with cancer stage and clinical outcomes. The results suggest the potential role of OATP in cancer development and progression and their potential role as new targets in cancer treatment [10, 12, 17, 20, 28, 34, 35]. Recently, Chen et al*.* showed that the OATP1B3 expression was significantly reduced in neoplastic tissues compared to that in adjacent non-neoplastic tissues. Moreover, the OATP1B3 lower expression was significantly correlated with the tumor size, relapse, tumor differentiation, and tumor node metastasis (TNM) rate in hepatocellular carcinoma [36].

The expression, substrate specificity, and activity of OATP transporters in tumors may affect the intracellular concentration of drugs, and, therefore, influence their effectiveness. OATP1B1 mediates hepatic uptake of many drugs and can influence transporter-mediated drug-drug-interactions (DDIs), therefore is responsible for the multiple side effects of multi-drug therapy, often used in cancer treatment [37]. Furthermore, expression levels of these influx transporters may play a crucial role in chemoresistance mechanisms [13]. Patients with OATP polymorphisms have been found to have altered pharmacokinetics due to their impact on absorption, distribution, and excretion of anticancer drugs, thus cancer outcomes [11, 20, 21, 38]. Bortezomib as a proteasome inhibitor is used in multiple myeloma treatment. Alam et al. in an in vivo study investigated that bortezomib has a low potential to cause OATP-mediated clinical drug-drug interactions (DDIs) [39].

The availability of results on the role of polymorphisms in these important transporters in cancers is limited, especially in the case of hematologic malignancies. Some single nucleotide polymorphisms (SNPs) in the genes encoding OATPs have been reported to be clinically relevant and have been investigated for their impact on the expression, and protein function. Only several are known to have functional effects and clinical significance, e.g. SLCO1B1 rs2306283 (A388G, N130D) or rs4149056 (T521C, V174A) [16, 17, 26, 28]. The A388G and T521T C form four main haplotypes: *1A (388A/521T)—wild-type, *1B (388G/521T), *5 (388A/521C) and *15 (388G/521C) [11, 15]. The clinical importance of SLCO1B1, mainly *5 or *15, for statin-induced myopathy is well demonstrated [39]. The A388G SNP was reported with the altered transport function caused by changes in the structure of the protein’s transmembrane-spanning domains [28].

The G allele at A388G (referred to as the *1B variant) causes a substitution that may increase the OATP1B1 activity, however, role of this variant remains moot [30]. Studies on the functional consequences of the * 1B haplotype have been partially controversial as some studies have shown reduced activity, others have shown increased activity, and many have shown no change in transport activity [40]. Absolute protein quantification showed that OATP1B1 protein levels were significantly higher in the GG genotype vs. the AA genotype in A388G SNP, confirming the increased transport function of N130D-OATP1B1 in vivo [11, 31]. These results can confirm that the presence of the G allele influencing the increase in the expression of the protein responsible for the intracellular transport of chemotherapeutic agents leads to more efficient transport and a higher concentration of the drug in the cell, which makes the drug therapy more effective. Some studies revealed that OATP1B1 could enhance the transport of drugs by transporters, and an in vivo experiment reached the same conclusion [15, 41]. Therefore, study aimed to assess the potential impact of the one of most common functional A388G SNP variants in *SLCO1B1* gene on the risk of multiple myeloma development and clinical outcomes.

The discrepancy in some results may be due to the differences in ethnicity, as *SLCO1B1* allele frequencies are known to vary between different populations [28]. In our research, the G allele prevalence was close to the frequencies reported in other Caucasian populations: 39.2% in the multiple myeloma group and 36.9% in the control group. The obtained results are consistent with the data published by Nagy et al*.* where the frequency of the G allele in the A388G SNP of the *SLCO1B1* gene was 36.2% in Hungarian populations [26].

The study showed a statistically significant association between the GG genotype with longer survival of patients with multiple myeloma with MP therapy compared to other treatment regimens (*p* = 0.0271). Statistical significance was found also for the presence of at least one A allele and age at diagnosis ≤ 63 years (*p* = 0.0357)*.* These are only the results of statistical analysis between two subgroups of patients with MM. However, it may potentially indicate that despite the presence of the A—wild allele theoretically associated with the normal (protective) function of the protein, patients got sick anyway for multiple myeloma in the subgroup of younger patients (< 63 years of age) who, due to their younger age, were in a group with a potentially lower risk of developing the disease. The study showed that the AA genotype and the A allele were more common in the control population, while the GG genotype and the G allele were more common in multiple myeloma patients. However, the obtained results did not show a statistically significant association between the studied polymorphism and the risk of multiple myeloma (*p* = 0.8211). In addition, no significant association with clinical-pathological features was found.

The studied polymorphism has not been verified in multiple myeloma or other hematologic neoplasms so far. Therefore, we are not able to relate our results to other studies in MM. Our results can only be compared with those obtained in studies on solid tumors. Falkowski et al. have shown that the A388G variant genotypes of *SLCO1B1* were not associated with colorectal cancer (CRC); similar results were obtained by Özhan et al*.* in colorectal cancer [21, 42]. In another study on two common polymorphisms of *OATP4A1*, no association between CRC predisposition and tumor recurrence was found [38]. Noci et al*.* showed that A388G is associated with overall survival for patients with metastatic CRC treated with irinotecan. Previously in Innocenti et al*.* study, this SNP has been reported to be associated with neutropenia in patients treated with irinotecan [29].

In the presented study, the dependence of the A388G in the *SLCO1B1* gene on the probability of overall survival time has been assessed. The OS was longer if the G allele was present in the genotype, however, there was no statistically significant difference in survival according to genotypes or alleles. Our results were consistent with those obtained by Zhang X. et al., which showed no difference in overall survival between wild-type and SLCO1B1 A388G carriers in breast cancer patients [43]. On the contrary, Teft et al. have found that progression-free survival (PFS) was significantly longer in *SLCO1B1* 388G/G colorectal cancer patients after irinotecan-based chemotherapy [44].

Most of the research has been devoted to the role of transporters, polymorphic variants, and haplotypes in the pharmacokinetics of drugs, including chemotherapeutic agents used in the treatment of hematological malignancies [45–47]. A recent study in adult patients with hematologic malignancies receiving high-dose methotrexate suggests that patients with the *SLCO1B1* A388G or T521C variants exhibit differential metabolomic profiles that may modulate the risk for methotrexate-induced toxicities. Similar findings have been reported in cancer patients treated with irinotecan, the plasma concentration of active metabolite SN-38 was higher and the risk of severe neutropenia was increased by T521C, while the A388G variant does not affect transport activity for SN-3. The results obtained in the studies indicate that the importance of OATP transporters is worthy of further attention from the researchers [10, 47, 48].

The expression, polymorphisms, substrate spectrum, importance in drug transport, DDIs, and multi-drug resistance mechanisms, turn out to be not the only interesting OATP application in medicine [49]. Zhang et al*.* presented a next different view on the usefulness of OATP transporters in cancer. They have shown that due to the overexpression of OATPs in many types of cancer cells and their active transport function, the diagnostic substance can more effectively penetrate the cell membranes of cancer cells, unlike healthy cells. The results may contribute to the development of a promising diagnostic tool for the differentiation of cancer cells in the early stages of diagnosis [50].

Despite the enormous advances in MM treatment in recent decades and the availability of new drugs and their combinations, including new generations of proteasome subunit inhibitors, immunomodulatory drugs or antibodies with incorporating autologous stem cell transplantation (ASCT), MM is still an incurable disease that requires lifelong management. The authors are aware of the limitations of the results presented in the study. The results presented in the study do not fully correspond to the currently used therapy standards, prognostic indicators, such as International Staging System (ISS) or Revised International Staging System (R-ISS) or more appropriate indicators for the analysis of survival, such as progression-free time or time to progression, but not only overall survival. However, the study limitations mainly result from the fact that the material used in the study was collected in the years 1992–2002 and also the limitations of access to complete clinical data and laboratory results, which the authors had no influence.

Increased studies are necessary to obtain more comprehensive profiles of OATPs differentially regulated in cancer cells and further investigate the role of OATPs in multiple myeloma. This will allow researchers to better understand molecular mechanisms underlying an altered expression of OATPs in hematologic cancer development, anticancer drug transport, and therapy efficiency to determine how these transporters can be used as potential molecular markers. Further analyzes of polymorphic variants in OATP transporters are planned, including haplotype analyzes, effects on protein expression, and function. Nevertheless, the authors believe that the presented manuscript brings new information about the share of polymorphism in the Polish population, which has not been studied so far in this disease entity.

## Conclusion

According to our knowledge, this is the first such study in multiple myeloma patients, especially in the Polish population. Our study showed that SNP A388G of the *SLCO1B1* gene does not predispose to an increased individual risk of developing multiple myeloma and does not affect the values of selected clinical parameters. However, the occurrence of the GG genotype may prolong patient’s overall survival in the case of Melphalan-Prednisone therapy. Due to contradictory reports on the importance of this polymorphism on the amount and function of the protein, an in silico analysis was performed which did not show the effect of SNP on the structure and function of the protein. Further studies are necessary to obtain more comprehensive profiles of OATPs differentially regulated in cancer cells, along with a better understanding of molecular mechanisms underlying the altered function of OATPs in cancer.

## Data Availability

The data presented in this study are available on request from the corresponding author.
